# Bayes or determinables? What does the bidirectional hierarchical model of brain functions tell us about the nature of perceptual representation?

**DOI:** 10.3389/fpsyg.2012.00500

**Published:** 2012-11-15

**Authors:** Bence Nanay

**Affiliations:** ^1^Centre for Philosophical Psychology, University of AntwerpAntwerp, Belgium; ^2^University of CambridgeCambridge, UK

**A commentary on**

**Whatever next? Predictive brains, situated agents, and the future of cognitive science**

by Clark, A. (in press). Behav. Brain Sci.

Andy Clark has a mission. He has been attaching the classical/computationalist concept of representation for almost 25 years, always from different angle and always with the purpose of salvaging the concept of representation from the mistaken classical/computationalist assumptions. Unlike the new generation of anti-representationalists and enactivists who want to reject the concept of representation altogether, he wants to keep it, but he also wants to strip it from a wide variety of computationalist garments [this is the most explicit in Clark, 1995, 1997, Chapter 8]. The target article is his newest attempt to do so.

Clark's argument in this paper proceeds in two steps:

- It uses empirical data to support a theoretical model of the mind, the “bidirectional hierarchical model of brain functions” whereby “a cascade of top-down processing generates low-level states from high-level causes” (p. 25).
- It uses this theoretical model to draw some philosophical conclusions.

I accept step A and focus on step B. My aim is to argue that even if we accept that the empirical data Clark considers does support the “bidirectional hierarchical model of brain functions,” what he takes to be his most interesting philosophical conclusion would still be problematic.

More specifically, Clark makes three strong philosophical claims about how we should revise our philosophical theories of the mind in general and the concept of representation in particular in the light of the empirical evidence that supports the “bidirectional hierarchical model of brain functions”:

- The “bidirectional hierarchical model of brain functions” shows that the distinction between perception and cognition is blurred.
- A salient feature of the “bidirectional hierarchical model of brain functions” is that the “high-level” representation is not about the present perceived scene, but about what the perceived scene will be when the “low-level” input is processed. This means that this “high-level” represents a future state of affairs.
- The “bidirectional hierarchical models of brain functions” shows that perceptual representations represent probabilities (or, represent probabilistically, or, represent probability density distributions—Clark does not always distinguish these three interpretations).

I agree with Clark that (a) and (b) are very important philosophical consequences that no philosophical account of the mind should ignore (see, e.g., Nanay, 2011a, 2012, 2013, forthcoming). The focus of this commentary is what Clark takes to be the most groundbreaking of the philosophical import of the “bidirectional hierarchical model of brain functions,” namely, the claim that perceptual representations represent probabilities. This is what makes his account Bayesian and this is a philosophical or theoretical conclusion that neuroscientists and psychologists are also quick and happy to draw.

My claim is that nothing in the “bidirectional hierarchical models of brain functions” implies that perceptual representations are probabilistic, or that they represent or “encode probability density distributions” [p. 21, see also Knill and Pouget (2004), p. 713]. There is a much more parsimonious way of describing the representations in the bidirectional hierarchical model of brain functions: they attribute properties to objects (or to the perceived scene) that are not fully determinate (Yeshurun and Carrasco, 1998; Nanay, 2010, 2011b, 2013; Stazicker, 2011).

Properties can be more or less determinate. To use a classic example, being red is more determinate than being colored, but less determinate than being scarlet (Funkhouser, 2006). When a representation attributes a property to an object, this property can then be more or less determinate. Representing something as red or representing it as scarlet (or a specific shade of scarlet) would amount to different representations.

As long as we do not take perceptual representations to attribute fully determinate properties, we do not need to posit probabilistic representations. Rather than conjecturing that perceptual representations are probabilistic or that they encode probability density distributions, we can describe them in a much less controversial manner: they attribute less than fully determinate properties. To use a very simple example, they do not represent the probability density distribution of various (fully determinate) shades of scarlet, ruby, and crimson; they merely represent the less than fully determinate (or, as some philosophers say, determinable) property of red.

We have plenty of independent empirical evidence for this way of thinking about perceptual representations: we know that our perceptual system cannot attribute highly determinate color properties to the periphery of our visual field and the inattentional and change blindness experiments suggest that our perceptual system does not even attribute fully determinate properties to more central parts of the perceived scene.

But if this is so, then we can use a similar move when it comes to the bidirectional hierarchical model of brain functions: the “top level” in this model could also be interpreted as representing the perceived scene as having not fully determinate properties that the “bottom level” then makes more determinate. Nothing in this way of cashing out the bidirectional hierarchical model of brain functions implies either that these representations are probabilistic or that they represent “probability density distributions.” The changes in the determinacy of the properties attributed by these representation may follow Bayesian rules, but the representations themselves neither represent probabilities nor represent whatever they represent probabilistically.

Clark's oeuvre can be split into two very different streams when it comes to criticisms of the classical/computationalist concept of representation. On the one hand, he urges philosophers and cognitive scientists to work with a concept of representation that is simpler, less over-intellectualized, and more similar to the representational capacities of simple animals (Clark, 1989, 1995, 1997, 2009). One the other hand, he emphasizes uniquely human, highly complex and not at all animal-like aspects of our representational system (Clark and Chalmers, 1998; Clark, 2008). The present article clearly belongs to the former group: it emphasizes an aspect of the mind that is present both in animals and in humans. But then shouldn't we opt for the least over-intellectualizing, most parsimonious concept of the representations in the bidirectional hierarchical model of brain functions? If we do, these representations will have nothing Bayesian about them.
